# Radiomics features of computed tomography and magnetic resonance imaging for predicting response to transarterial chemoembolization in hepatocellular carcinoma: a meta-analysis

**DOI:** 10.3389/fonc.2023.1194200

**Published:** 2023-07-13

**Authors:** Lijuan Feng, Qianjuan Chen, Linjie Huang, Liling Long

**Affiliations:** ^1^ Department of Radiology, First Affiliated Hospital of Guangxi Medical University, Nanning, Guangxi, China; ^2^ Key Laboratory of Early Prevention and Treatment for Regional High Frequency Tumor, Gaungxi Medical University, Ministry of Education, Nanning, Guangxi, China; ^3^ Guangxi Key Laboratory of Immunology and Metabolism for Liver Diseases, First Affiliated Hospital of Guangxi Medical University, Nanning, Guangxi, China

**Keywords:** hepatocellular carcinoma, radiomics, transarterial chemoembolization, tumor response, systematic review

## Abstract

**Purpose:**

To examine the methodological quality of radiomics-related studies and evaluate the ability of radiomics to predict treatment response to transarterial chemoembolization (TACE) for hepatocellular carcinoma (HCC).

**Methods:**

A systematic review was performed on radiomics-related studies published until October 15, 2022, predicting the effectiveness of TACE for HCC. Methodological quality and risk of bias were assessed using the Radiomics Quality Score (RQS) and Quality Assessment of Diagnostic Accuracy Studies-2 tools, respectively. Pooled sensitivity, pooled specificity, and area under the curve (AUC) were determined to evaluate the utility of radiomics in predicting the response to TACE for HCC.

**Results:**

In this systematic review, ten studies were eligible, and six of these studies were used in our meta-analysis. The RQS ranged from 7-21 (maximum possible score: 36). The pooled sensitivity and specificity were 0.89 (95% confidence interval (CI) = 0.79–0.95) and 0.82 (95% CI = 0.64–0.92), respectively. The overall AUC was 0.93 (95% CI = 0.90–0.95).

**Conclusion:**

Radiomics-related studies evaluating the efficacy of TACE in patients with HCC revealed promising results. However, prospective and multicenter trials are warranted to make radiomics more feasible and acceptable.

## Introduction

Hepatocellular carcinoma (HCC) is a type of liver cancer that is responsible for a significant number of cancer-related deaths globally, ranking as the third highest cause, with a relative 5-year survival rate of approximately 18%, thus posing a heavy health burden globally (1, 2). Transarterial chemoembolization (TACE) is the first-line treatment method in patients with intermediate-stage HCC and the most widely used treatment method for unresectable HCC (3, 4). TACE can produce survival benefits and favorable responses without causing adverse effects on hepatic functional reserve, if performed correctly (5). Owing to the high heterogeneity of HCC, it is difficult to achieve a satisfactory tumor response from a single session of TACE (6). Therefore, to enhance the effectiveness of TACE and the overall survival rate, prompt and precise identification of suitable candidates for the treatment is crucial (7).

Predicting the tumor response to TACE and the overall survival rate can contribute to achieving this goal. The hepatoma arterial embolization prognostic (HAP) score by Kadalayil et al. (8), the modified HAP-II score by Kim et al. (9), and the modified HAP-III score by Cappelli et al. helped in predicting outcomes in patients treated with TACE, but these scores were HCC-specific rather than TACE-specific (10). A combination of the STATE score by Hucke et al. (11) and the Assessment for Retreatment with TACE score can identify the best candidates for TACE. However, none of the aforementioned scoring systems are widely used in clinical practice (12, 13).

Radiomics involves extracting quantitative features from standard medical imaging using high-throughput mining techniques and is gaining importance in cancer research, as the data obtained using radiomics can be applied in clinical decision support systems to improve diagnostic, prognostic, and predictive accuracy (14–17). Hence, radiomics is widely used in the management of HCC, with a growing interest in employing it to predict the efficacy of TACE in patients with HCC. Peng et al. (18) showed that computed tomography (CT)-based integration of radiomics and deep learning yielded excellent predictive performance, with an area under the curve (AUC) of 0.994 for predicting the response to TACE in validation cohorts. Further, Cannella et al. (19) suggested that magnetic resonance imaging (MRI)-based radiomics showed acceptable predictive performance, with an AUC of 0.791 for predicting the tumor response to TACE in 51 patients with HCC. At present, radiomics-related literature on HCC has identified various features and predictors for tumor response in several differently designed studies (multicenter versus single center), image segmentation methods (manual versus semi-automatic segmentation), imaging modality (CT or MRI), and predictive models (hand-crafted radiomics versus machine learning versus deep learning methods) (20). However, researchers have not yet reached a consensus on the most effective means to use radiomics to predict treatment response in patients with HCC who undergo TACE. Nevertheless, the Radiomics Quality Score (RQS) proposed by Lambin et al. in 2017 promotes standardized data collection, evaluation criteria, and reporting guidelines (16).

Therefore, this study aimed to evaluate the methodological quality of radiomics-related studies and analyze the effectiveness of TACE in HCC. We also performed a meta-analysis to investigate the ability of radiomics to predict treatment response to TACE for HCC.

## Materials and methods

### Literature retrieval

This study was conducted in adherence to the Preferred Reporting Items for Systematic Reviews and Meta-Analyses for Diagnostic Test Accuracy (PRISMA-DTA) guidelines (21).

To identify relevant studies, PubMed, Embase, Cochrane Library, and Web of Science databases were searched independently by two observers. The search was performed on October 15, 2022, without any start-date limit, and was limited to publications in English. The search terms were (“radiomics” OR “texture” OR “textural” OR “histogram”) AND (“transcatheter arterial chemoembolization” OR “transarterial chemoembolization” OR “TACE”) AND (“hepatocellular carcinoma” OR “HCC”). Screening studies were performed using Endnote software version X9. Disagreements were resolved by consensus.

### Study screening

Two reviewers (LJ, F and LJ, H) screened the titles and abstracts of potentially relevant studies for their appropriateness. Inconsistencies were discussed by the reviewers (Ll, L), and a consensus was reached.

The inclusion criteria for articles were: (1) use of radiomics to predict the objective response (complete or partial response) and nonresponsiveness (stable disease or progressive disease); (2) imaging examination (CT and MRI) performed before the first TACE and availability of imaging findings after the first TACE; (3) the modified response evaluation criteria in solid tumors(mRECIST) used to assess tumor response after TACE; and (4) studies that could be obtained with the full text in English. The exclusion criteria for articles were: (1) case reports or case series including five or fewer patients; (2) conference papers, abstracts only, reviews, and letters; (3) studies involving the same patients; and (4) research on topics of interest other than the response to TACE in HCC.

### Evaluation of tumor response criteria for TACE

The assessment of tumor response to TACE in all the included articles was based on mRECIST (22). Complete tumor response and partial tumor response were defined as objective responses to TACE treatment, whereas stable disease and disease progression were defined as non-response to TACE treatment. Based on mRECIST, the objective response rate was utilized as the criterion to identify beneficiaries among patients with HCC in this meta-analysis, which primarily focused on assessing the predictive value of radiomics in the post-TACE response rate.

### Data extraction

The relevant information, which served as the largest AUC in the validation cohorts (testing cohorts), was extracted. The calculation of the pooled AUC required the standard error of the AUC; in cases wherein the value was not obtained in the literature, we used the formula proposed by Hanley and McNeil (23). If an external verification dataset did not exist, the results from the internal verification dataset set were employed. In cases where the internal verification dataset was not available, the results from the training (development) dataset were selected. The collected models contained basic information (such as publication year, first author, sample size, and research type) and radiologically relevant characteristics, such as radiomics information and image parameters.

### Quality assessment

The RQS and Quality Assessment of Diagnostic Accuracy Studies (QUADAS-2) were applied to evaluate the utility of radiomics research and the methodological quality of the included studies, respectively (16, 24). By analyzing data selection, medical imaging, feature extraction, exploratory analysis, together with modeling that includes 16 important components, the RQS evaluates the value of radiomics research. Details about RQS are available in the **Supplementary Material**. The RQS comprises 16 important components, each of which is assigned a score based on its importance in assessing the methodological quality of the study. The total score ranges from -8 to +36 points, with scores of -8 to 0 points corresponding to 0% and 36 points corresponding to 100%. The QUADAS-2 evaluates the bias risk in “Patient Selection,” “Index Test,” “Reference Standard,” and “Flow and Timing” using Review Manager 5.4 in or deter (24).

### Statistical analysis

We conducted a meta-analysis to predict the treatment response to TACE in patients with HCC. Two independent reviewers extracted the data, and a third reviewer assessed the internal validity.

Only studies that provided a 2×2 contingency table or sufficient information for reconstruction were included. If multiple models were present, only the model with the largest AUC was selected.

The pooled AUC, sensitivity, and specificity were used to quantify the value of radiomics based on CT and MRI findings to predict the tumor response to TACE in patients with HCC. Forest plots and summary receiver operating characteristic curves were obtained. Publication bias was assessed using funnel plots. The threshold effect was assessed by calculating the sensitivity and specificity using Spearman’s correlation coefficients. Cochran’s Q test and the I^2^ index were used to evaluate heterogeneity among eligible studies. An I^2^ value of <50% indicates low to moderate heterogeneity between studies and >50% indicates high heterogeneity.

IBM SPSS Statistics (version 26.0; IBM Corporation, Armonk, NY, USA), Stata (version 15.1), and Review Manager (version 5.4) were used for the statistical analyses. Statistical significance was set at P <0.05.

## Results

### Literature search

A total of 243 relevant articles were obtained. The titles and abstracts of 137 articles were assessed after removing of duplicate publications, and 94 articles were excluded.

**Figure 1:**
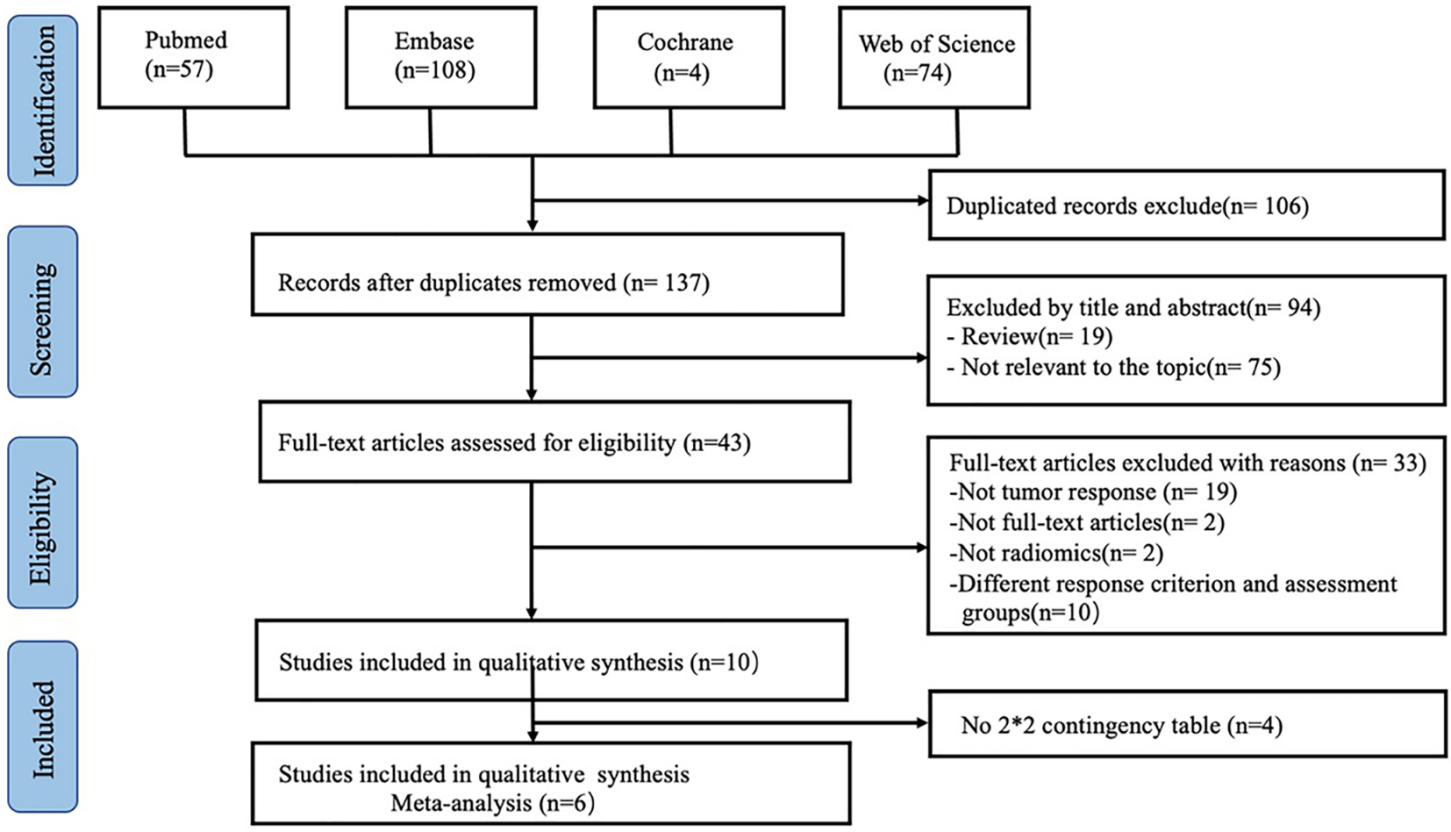
Flowchart of article selection in this study.

Forty-three relevant articles were screened for further analysis. After the selection process, ten articles were selected for qualitative meta-analysis (13, 18, 19, 25–31). Four articles that predicted tumor response did not supply sufficient information to rebuild a 2×2 table and evaluate the overall outcome (26–28, 31). Finally, six articles were included in our meta-analysis (13, 18, 19, 25, 29, 30). The article selection flowchart is shown in **Figure 1**. The characteristics of the included studies are presented in **Table 1**. The ten studies included the use of radiomics, with three studies based on CT and three on MRI.

**Table 1:** Characteristics of the included studies.

Author	Country	Study design	Duration of patient recruitment	Center	TRIPOD type	Training dataset	Test dataset	Mean,age, (year)	Male,n (%)	Imaging modality	Imaging sequences
Chen 2021	China	Retrospective	2010–2014	M	Type 3a	355	122	56	113 (93.4)	CT	CECT, NC-CT
Kong 2021	China	Retrospective	2016–2019	S	Type 2a	69	30	NA	NA	MRI	T2WI
Kuang 2021	China	Retrospective	2014–2019	M	Type 3a	113	40	64	32 (80.0)	MRI	T2WI, DCE-MR AP
Mao 2021	China	Retrospective	2018–2019	S	Type 1a	45	0	62	37(82.2)	CT	CECT
Peng 2021	China	Retrospective	2015–2020	M	Type 3a	139	171	NA	152(88.9)	CT	CECT
Zhao 2021	China	Retrospective	2008–2019	S	Type 2a	85	37	60	32 (86.5)	MRI	AP, PVP, DP
Liu 2022	China	Retrospective	2013–2019	M	Type 3a	94	46	NA	NA	MRI	T2WI, AP, PVP, DP, Mp-MRI
Guo 2022	China	Retrospective	2018–2020	S	Type 2a	47	47	58	40 (85.1)	CT	NC-CT
Bai 2022	China	Retrospective	2016–2019	S	Type 2a	79	32	59	22(68.8)	CT	AP, PVP,
Cannella 2022	Italy	Retrospective	2015–2020	S	Type 1b	51	0	73	37 (72.5)	MRI	PVP, 3′transitional, HBP
Author	Segmentation method		ROI	ROI Software	Feature Extraction		Features Type		Feature selection		Modeling method
Chen 2021	semiautomatic segmentation		T+P	3D slicer	3D slicer, PyRadiomics package		shape, GLDM, GLCM, First order, GLRLM GLSZM, NGTDM, Wavelet		LASSO		multivariate analysis backward step-down process
Kong 2021	NA		T	ITK-SNAP	Artificial Intelligence Kit software		form factors, histogram, GLSZM, GLCM, RLM		LASSO		Univariate analysis and multivariate analysis
Kuang 2021	semi-automatically		T	ITK-SNAP	Artificial Intelligence Kit software		histogram feature, morphological, GLSZM, GLCM, RLM		mRMR, LASSO		Univariate and multivariate logistic regression
Mao 2021	Manual delineation		T	CT-Kinetics program	NA		IBS, IBH GLCM, GLRLM		ICC, SCC, univariate logistic regression, LASSO		multivariate logistic regression, CCA, DCA
Peng 2021	Manual delineation		T	ITKSNAP, MATLAB 2014b	Python		Wavelet transform, original_ngtdm_Complexity, logarithm_ngtdm_Busyness		RFE, 5-fold cross-validation		Linear logistic, SVM, GBM, RF
Zhao 2021	Manual delineation		T	ITK-SNAP	A. K. software		histogram, GLCM, GLRLM, GLZSM, Haralick, form factors, Gaussian transform features.		ICC, SCC, univariate logistic regression, LASSO		multivariate logistic regression algorithm
Liu 2022	Manual delineation		T	ONCO IMAG ANLY, Python	Python Pyradiomics		First order intensity, Shape, GLSZM, GLRLM, NGTDM, GLDM, GLCM, Log-sigma, Wavelet		mRMR, LASSO		univariate and multivariate logistic regression (LR) analyses
Guo 2022	NA		T	NA	MaZda		Histogram, image Gradient, RLM, Wavelet transform		logistic regression analysis, Lasso		Lasso , six twelve grade, stepwise regression
Bai 2022	Manual delineation		T	MITK	PyRadiomicss		Shape, histogram, Wavelet		ICC, SCC, mRMR, WLCX		RF, SVM, LASSO
Cannella 2022	Manual delineation		T	Radiomics	PyRadiomics		histogram, GLCM, GLDM, GLRLM, GLZLM, NGTDM, shape, wavelet		LASSO, point-biserial correlation coefficient		logistic regression

Center: S single center, M multi-center: Imaging modality: US ultrasound, CT computed tomography: MRI magnetic resonance imaging: Imaging sequences: CECT contrast enhanced computed tomography, NC-CT non-contrast, T2WI T2-weighted imaging, DCE-MRI dynamic contrast-enhanced MRI arterial phase, PVP portal venous phase, DP delayed phase, Mp-MRI Multi-parametric MRI, 3′transitional 3-min transitional, HBP hepatobiliary phases; Features Type: GLCM gray level co-occurrence matrix, GLRLM gray level run length matrix, GLSZM grey-level zone size matrix, GLDM gray level dependence matrix: NGTDM neighborhood gray-tone difference matrix, RLM run length matrix, IBS intensity-Based Statistical, IBH intensity-Based Histogram; Feature selection: CNN convolutional neural network, GBRT gradient boosted regression trees, LASSO least absolute shrinkage and selection operator, mRMR maximum correlation–minimum redundancy, RFE: Recursive feature elimination, SCC Spearman correlation coefficients, WLCX Wilcoxon rank-sum test, CCA Calibration Curves Analysis, DCA: Decision Curve Analysis, GBM gradient boosting machine, RF random forest, SVM support vector machine, AI Kit software Artificial Intelligence Kit software, RF the random forest; NA, Not Available.

### Quality of studies

The quality of each eligible article was assessed using the RQS, as shown in **Table 2**. The ten studies achieved a mean ± standard deviation RQS of 14.50 ± 4.55, a median of 14.50, and a range of 7–21. The mean proportion of the RQS was 40.28%, with a maximum value of 58.33%. The intra-class correlation coefficient (ICC) between the two independent reviewers who evaluated the articles was 0.940 (95% confidence interval (CI) = 0.888–0.975, p<0.001), indicating high reproducibility among reviewers. The RQS assessed by the two reviewers are demonstrated in **Tables S1** and **S2**.

**Table 2:** Radiomics Quality Score table of included studies.

Study	Chen et al	Kong et al	Kuang et al	Mao et al	Peng et al	Zhao et al	Liu et al	Guo et al	Bai et al	Cannella et al
Image Protocol (2)	2	1	1	1	1	1	2	0	1	1
Multiple Segmentations (1)	1	1	1	1	1	1	1	1	1	1
Phantom Study (1)	0	0	0	0	0	0	0	0	0	0
Multiple Time points (1)	0	0	0	0	0	0	0	0	0	0
Feature Reduction (3)	3	3	3	3	3	3	3	3	3	3
Non Radiomics (1)	1	1	1	1	1	1	1	1	0	1
Biological Correlates (1)	1	1	1	1	0	1	1	0	0	1
Cut-Off (1)	1	0	1	0	1	0	1	1	1	0
Discrimination/Resampling (2)	1	1	1	2	1	1	1	1	1	2
Calibration/Resampling (2)	1	1	1	1	0	1	1	0	0	0
Prospective (7)	0	0	0	0	0	0	0	0	0	0
Validation (5)	4	2	4	-5	4	2	3	2	2	-5
Gold Standard (2)	2	2	2	2	2	2	2	2	2	2
Clinical Utility (2)	0	2	0	2	0	2	2	0	0	0
Cost (1)	0	0	0	0	0	0	0	0	0	0
Open Science (4)	2	0	2	0	0	3	3	1	1	1
Total score (36)	19	15	18	9	14	18	21	12	12	7

Most studies provided details about the imaging protocols, performed multiple segmentation, conducted feature reduction, used multivariable analysis with applied discrimination statistics, and achieved their potential clinical utility. Validation of radiomics features in the independent validation cohort was performed in eight studies, and four studies used an external validation cohort. A few studies performed cut-off analysis, model calibration, and publicly shared segmentations or codes. None of the included studies used phantoms, were scanned at multiple time points, were prospective, or assessed cost-effectiveness.

**Figure 2:**
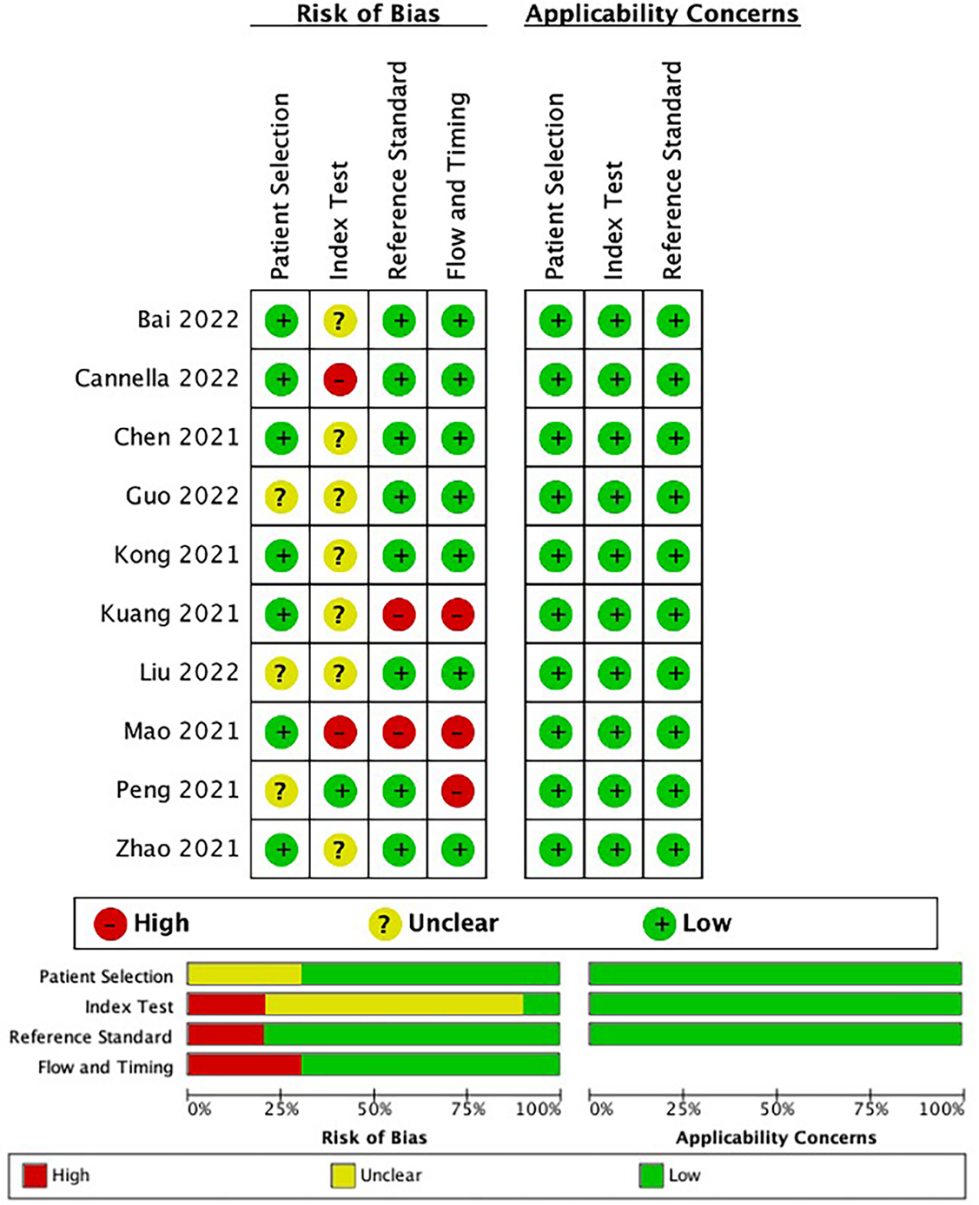
The bias risk and applicability using the Quality Assessment of Diagnostic Accuracy Studies (QUADAS-2) tool in this meta-analysis.

The risk of bias, as assessed using the QUADAS-2, is presented in **Figure 2**. In the patient selection domain, seven studies evaluated low bias risk and three assessed unclear risk. In the index test domain, one study had a low bias risk, and two studies had a high bias risk. Two and four studies received a high bias risk in the reference standard domain and fourth domain, respectively, and the remaining studies were considered to have a low bias risk in the last two domains. All studies in this meta-analysis had high applicability in the three domains. The details of the individual and final evaluations of the risk of bias and applicability concerns are presented in **Tables S3–S5**.

### Overall literature assessment

**Figure 3:**
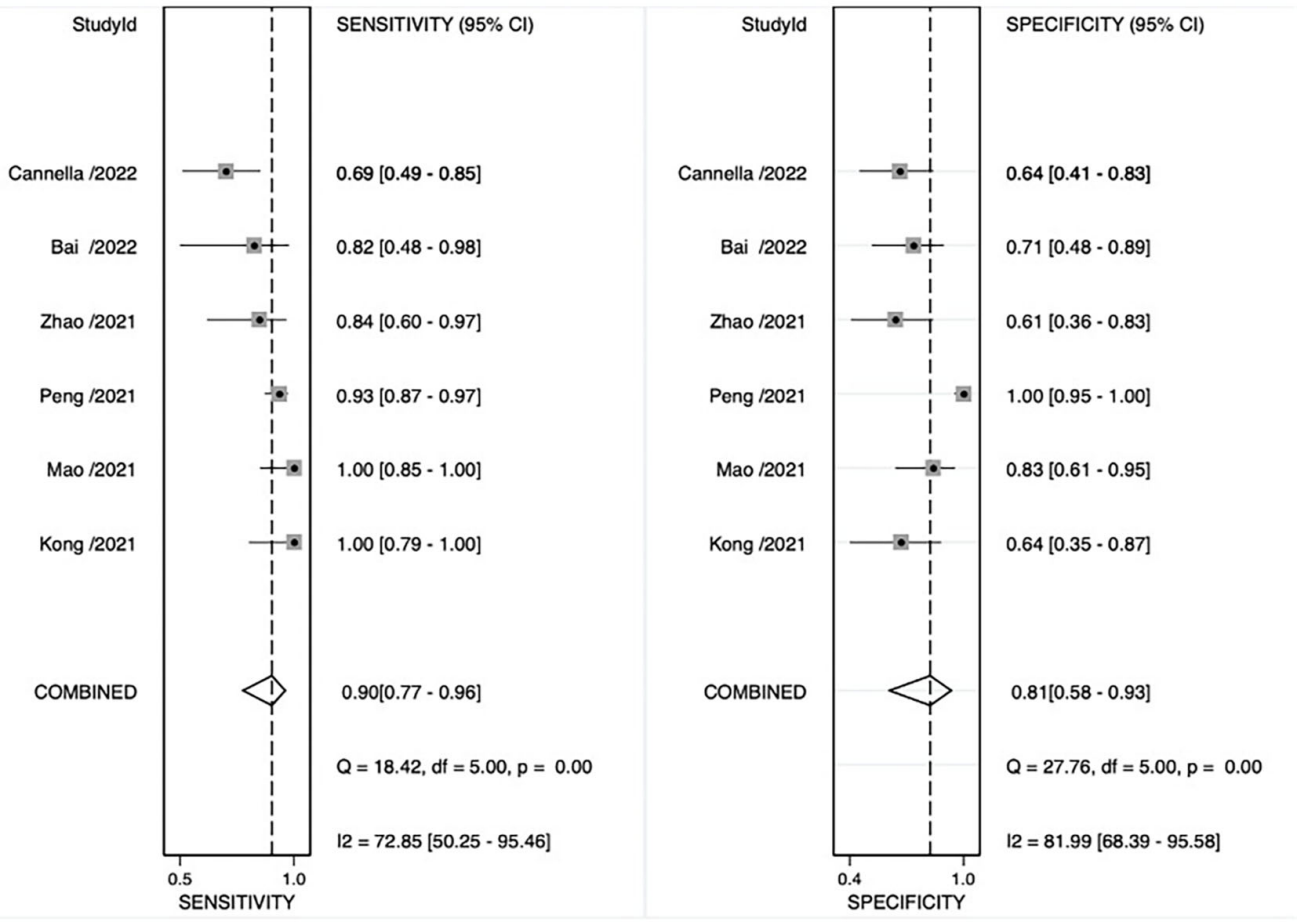
Coupled forest plot of sensitivity and specificity of radiomics models for preoperative prediction of response to transarterial chemoembolization (TACE).

**Figure 4:**
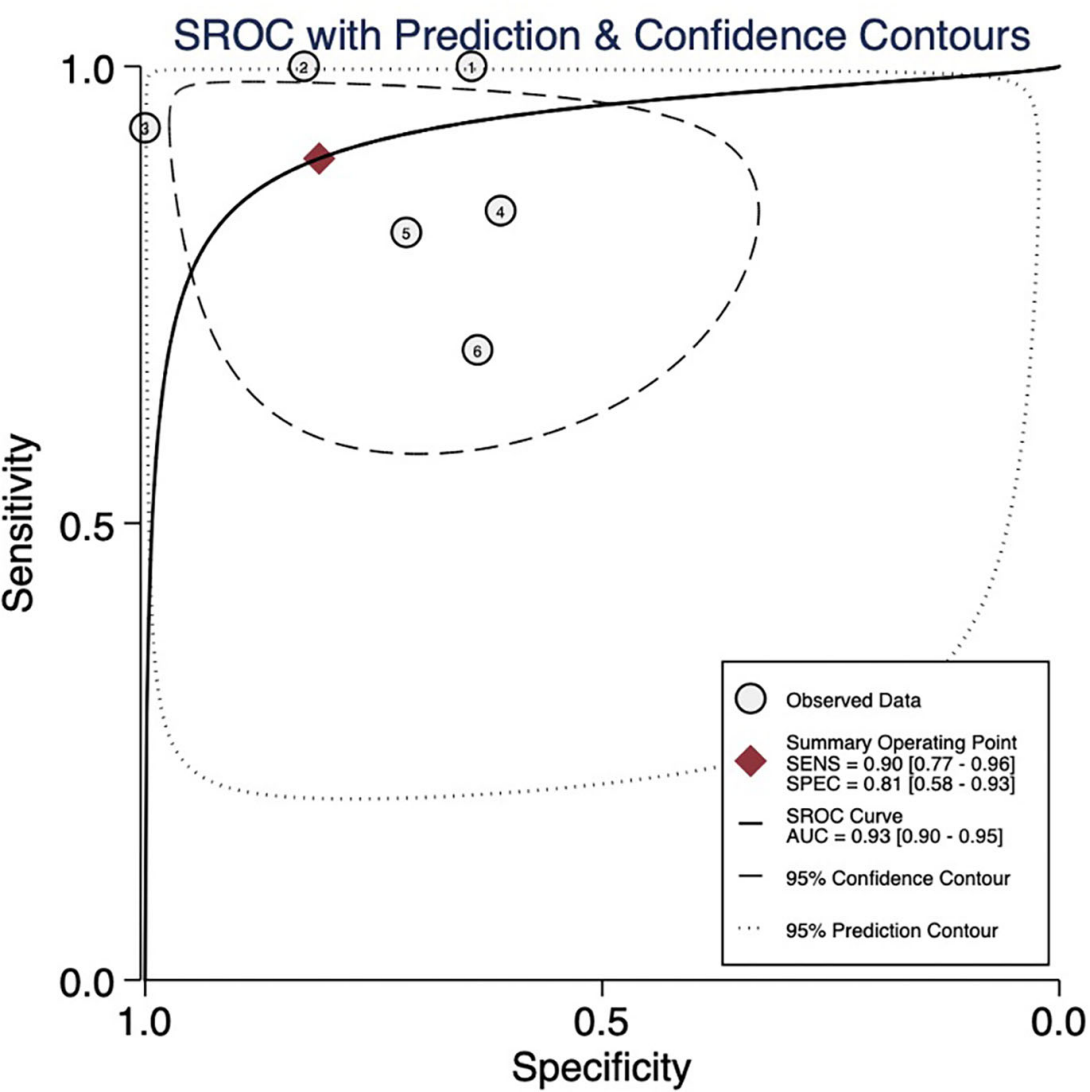
Summary receiver operating characteristic (SROC) curve. AUC, area under the curve.

**Figure 5:**
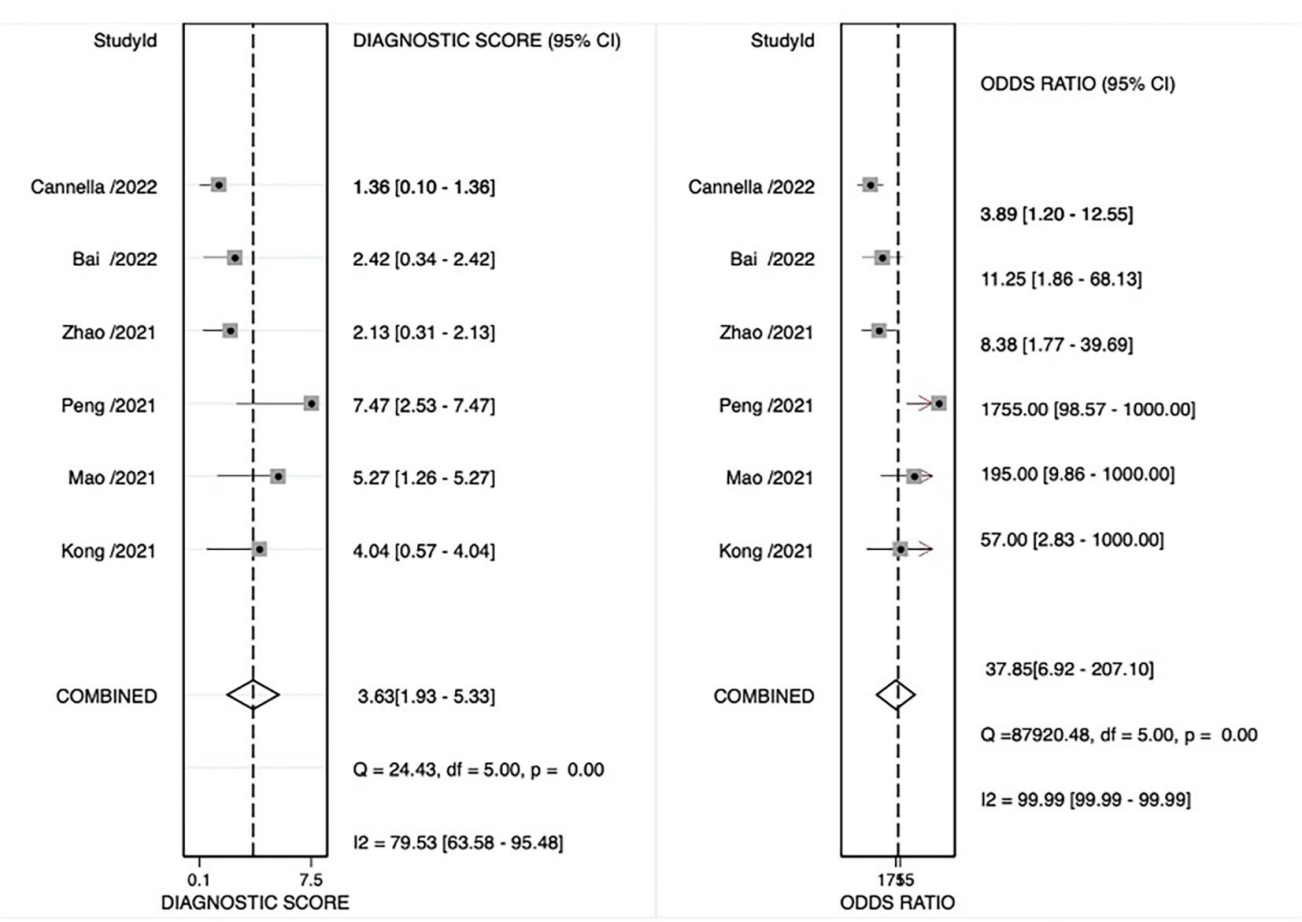
Odds ratio forest plot of radiomics models for preoperative prediction of response to transarterial chemoembolization (TACE).

**Figure 6:**
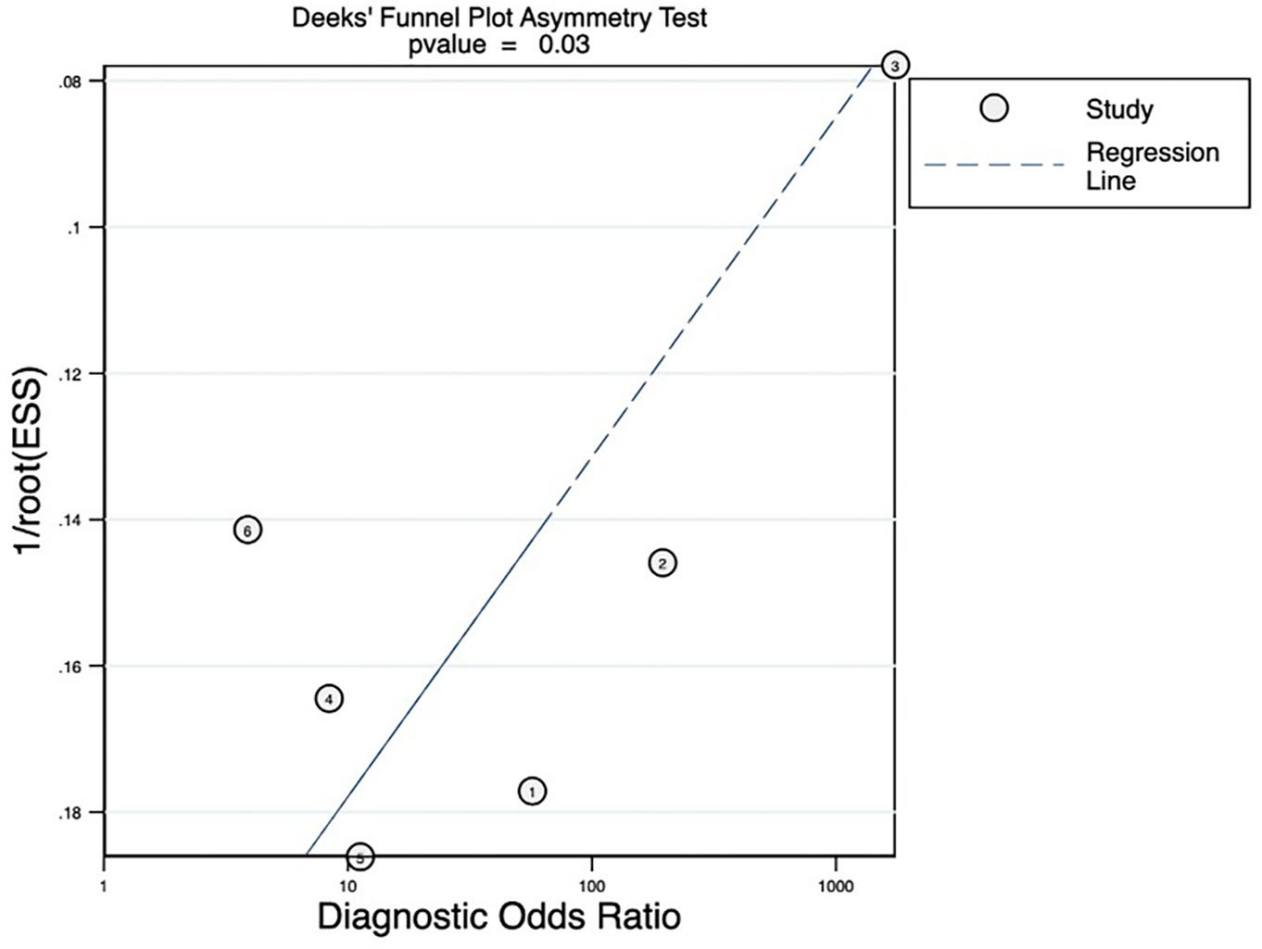
Deeks’ funnel plot revealing the possibility of publication bias, with a p value of 0.03. ESS, effective sample size.

The pooled sensitivity, pooled specificity, pooled AUC, and summary receiver operating characteristic (SROC) curves were used to evaluate the value of radiomics to predict the tumor response to TACE in patients with HCC. According to our data analysis, the pooled sensitivity and specificity were 0.90 (95% CI = 0.77–0.96) and 0.81 (95% CI = 0.58–0.93), respectively. The forest plot is shown in **Figure 3**. Significant heterogeneity in sensitivity (I^2 =^ 72.85%) and specificity (I^2 =^ 81.99%) was observed among eligible studies. The pooled AUC was 0.93 (95% CI = 0.90–0.95), as displayed in **Figure 4**. Three studies with radiomics based on CT indicated higher sensitivity (0.93 versus 0.81) and specificity (0.91 versus 0.63) than those with radiomics based on MRI. The random effects model was used to analyze the pooled diagnostic performance because of the high heterogeneity among the studies, and the results are shown in **Figure 3** and the diagnostic odds ratio in **Figure 5**. Deeks’ funnel plot (p=0.03) indicated publication bias, as shown in **Figure 6**. Furthermore, a Fagan plot was used to assess clinical utility. Using a radiomics model, the post-test probability increased to 83% from 50%, with a positive likelihood ratio of 5 when the pre-test was positive, and simultaneously, the post-test probability reduced to 11%, with a negative likelihood ratio of 0.12 when the pre-test was negative, as shown in **Figure S1**.

## Discussion

This systematic review and meta-analysis assessed the efficacy of radiomics in predicting tumor response to TACE for treating HCC.

Since the concept of radiomics was proposed, it has played an increasingly important role in the medical community, especially in the field of oncology, including disease diagnosis, pathological staging, and treatment evaluation. Despite the exponential growth in radiomics-related publications, routine clinical implementation is still lacking, indicating the need for a better understanding of the biological significance of any radiomic signature derived from radiomics (32, 33). The RQS has been proposed to assess the methodological quality of radiomics studies, which is important for critically appraising many publications and prioritizing the validation of high-quality results (34). Our independent RQS system was assessed by two independent reviewers, with a good level of agreement achieved in terms of the overall rating (ICC = 0.948).

The overall quality of this meta-analysis was relatively low (mean RQS = 14.50 ± 4.55, median ROS =14.50, range = 7–21). As noted in some meta-analyses (35–37), none of the included studies performed phantom studies, imaging at multiple time points, or cost-effectiveness analysis, perhaps because some of these methods may be difficult and not routine. All included studies were retrospective; hence, seven points were lost. In this review, the external validation was insufficient. Subsequently, multicenter validation and prospective studies are warranted to improve the reproducibility and stability of radiomics-based models. In studies of diagnostic models, the predictive performance of the model is usually assessed through calibration, discrimination, and classification measures (37). In this review, discrimination was assessed in all studies, wherein six studies (13, 25–27, 29, 31) evaluated the calibration, which was vital for determining the agreement between predictions and observed outcomes, and four studies (13, 25, 29, 31) evaluated the benefit for clinical application using decision curve analysis. Unfortunately, there was publication bias (p=0.03) in our meta-analysis. First, the publication bias of this meta-analysis may be caused by the small sample size effect. Second, there were differences in the basic characteristics of patients included in the literature. Finally, there is no standardized framework for the procedures and specifics of radiomics, resulting in a generally inadequate quality level.

In the patient selection domain, seven studies (13, 19, 25–27, 29, 30) reported well-documented image acquisition protocols, whereas four studies lacked clarity in patient selection and image acquisition protocols. In the index test domain, the risk of bias was mostly unclear, as reviewers might be aware of the presence of a response to TACE before tumor delineation. The reviewers were blinded to the results of the reference standard in one study (18), with a low risk of bias. Two studies (19, 25) without validation were assigned a high risk of bias. For the reference standard domain, two studies (25, 27) reported that the imaging methods for follow-up may be inconsistent with the methods prior to TACE, which may result in a high risk of bias. Regarding the fourth domain, three studies (18, 25, 27) were assigned a high bias risk because the imaging methods used for follow-up were inconsistent and did not clearly display inclusion and exclusion criteria.

In our review, the pooled analysis of the results was encouraging; nevertheless, they should be interpreted with caution because of the small sample sizes of the included studies and the fact that only a few articles were analyzed. Radiomics demonstrated potential for predicting tumor response in patients with HCC treated with TACE, with pooled AUC, sensitivity, and specificity of 0.93, 0.89, and 0.82, respectively. In our study, CT-based radiomics demonstrated higher sensitivity and specificity compared to MRI-based radiomics, possibly due to the thinner scan layer thickness in CT imaging, which allowed for capturing more information. Most of the studies included shape features, first-order features, and textural features simultaneously; the gray-level co-occurrence matrix, gray-level run-length matrix, gray-level size zone matrix, and run-length matrix are commonly used in textural features. Texture analyses depicting and objectively quantifying tumor heterogeneity are essential in identifying potential responders and non-responders (35). Eight studies used Wavelet.

One of the included studies (18) showed that a deep learning algorithm demonstrated higher predictive accuracy, with an AUC of 0.930 and 0.994. Radiomics characterizes images using hand-crafted quantitative features and mathematical formulas, which can describe the relationships between image pixels in a meaningful way; however, deep learning minimizes human input, seeking to discover patterns algorithmically (15). In the future, incorporating both deep learning and radiomics may produce further improvements in predictive performance owing to the analysis of previously unobserved relationships that are uncovered by deep learning (15). Only one study (26) segmented the tumor and peritumor, with an AUC of 0.900. Some studies have demonstrated that the tumor and peritumor radiomic signature can improve the prediction of early and late recurrence after liver resection (38) and have significance in predicting microvascular invasion (39) in HCC.

This review has some limitations. First, all studies included were retrospective in nature. Second, most of the studies included in this review had a relatively small sample size. Third, most of the included studies (9/10) were conducted in China, although HCC is a global public health challenge. Thus, the geographically imbalanced data may limit the generalizability of our findings. Fourth, the inclusion criteria for the literature search differed slightly. Finally, most of the studies included in this review lacked external validation, and their generalizability requires further verification. Hence, subsequent prospective and multicenter studies with large sample sizes should be conducted to improve the stability and reproducibility of predicting response to TACE in patients with HCC based on radiomics.

## Conclusion

Radiomics-related studies that explored the efficacy of TACE in patients with HCC have yielded promising results. However, prospective and multicenter trials should be performed in the future to make radiomics more feasible and acceptable.

## Author contributions

LF & QC: Conceptualisation and designing the study, writing the manuscript, analysing the results. LF & LH: Statistical analysis. LL: Verification of results, bias risk assessment, review of manuscript. All authors contributed to the article and approved the submitted version.
