# Supplementary material for: Radiomics features of computed tomography and magnetic resonance imaging for predicting response to transarterial chemoembolization in hepatocellular carcinoma: a meta-analysis
Source: Front Oncol. 2023 Jul 13;13:1194200. doi: 10.3389/fonc.2023.1194200 (PMC10374837; doi:10.3389/fonc.2023.1194200)
Supplement: Supplementary file 1 [file DataSheet_1.docx]

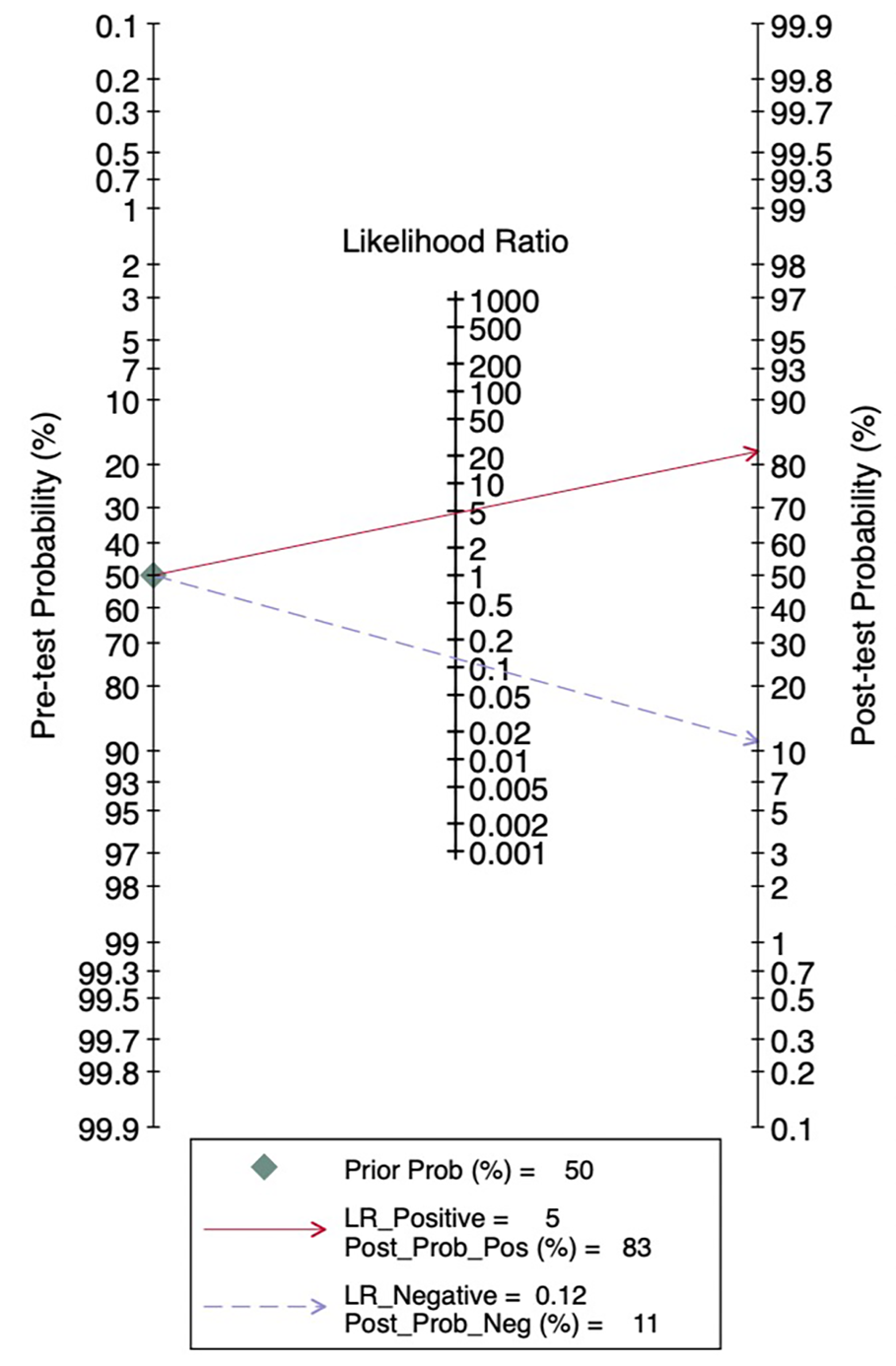


FigureS1 A Fagan plot.

Table S1 The RQS for review1

| ﻿Study | Chen et al | Kong et al | Kuang et al | Mao et al | Peng et al | Zhao et al | Liu et al | Guo et al | Bai et al | Cannella et al |
| --- | --- | --- | --- | --- | --- | --- | --- | --- | --- | --- |
| ﻿Image Protocol (2) | 2 | 1 | 1 | 1 | 1 | 1 | 2 | 0 | 1 | 1 |
| ﻿Multiple Segmentations (1) | 1 | 1 | 1 | 1 | 1 | 1 | 1 | 1 | 1 | 1 |
| ﻿Phantom Study (1) | 0 | 0 | 0 | 0 | 0 | 0 | 0 | 0 | 0 | 0 |
| ﻿Multiple Time points (1) | 0 | 0 | 0 | 0 | 0 | 0 | 0 | 0 | 0 | 0 |
| ﻿Feature Reduction (3) | 3 | 3 | 3 | 3 | 3 | 3 | 3 | 3 | 3 | 3 |
| ﻿NonRadiomics (1) | 1 | 1 | 1 | 1 | 1 | 1 | 1 | 1 | 0 | 1 |
| ﻿Biological Correlates (1) | 1 | 1 | 1 | 1 | 0 | 1 | 1 | 0 | 0 | 1 |
| ﻿Cut-Off (1) | 1 | 0 | 1 | 1 | 1 | 0 | 1 | 1 | 1 | 0 |
| ﻿Discrimination/Resampling (2) | 1 | 1 | 1 | 1 | 1 | 1 | 1 | 1 | 1 | 1 |
| ﻿Calibration/Resampling (2) | 1 | 1 | 1 | 1 | 0 | 1 | 1 | 0 | 0 | 0 |
| ﻿Prospective (7) | 0 | 0 | 0 | 0 | 0 | 0 | 0 | 0 | 0 | 0 |
| ﻿Validation (5) | 3 | 2 | 4 | -5 | 4 | 2 | 3 | 2 | 2 | -5 |
| ﻿Gold Standard (2) | 2 | 2 | 2 | 2 | 2 | 2 | 2 | 2 | 2 | 2 |
| ﻿Clinical Utility (2) | 0 | 2 | 0 | 2 | 0 | 2 | 2 | 0 | 0 | 0 |
| ﻿Cost (1) | 0 | 0 | 0 | 0 | 0 | 0 | 0 | 0 | 0 | 0 |
| ﻿Open Science (4) | 2 | 0 | 0 | 0 | 0 | 3 | 3 | 1 | 1 | 1 |
| Total score (36) | 18 | 15 | 16 | 9 | 14 | 18 | 21 | 12 | 12 | 6 |

Table S2 The RQS for review2

| ﻿Study | Chen et al | Kong et al | Kuang et al | Mao et al | Peng et al | Zhao et al | Liu et al | Guo et al | Bai et al | Cannella et al |
| --- | --- | --- | --- | --- | --- | --- | --- | --- | --- | --- |
| ﻿Image Protocol (2) | 0 | 1 | 1 | 2 | 1 | 2 | 2 | 0 | 1 | 2 |
| ﻿Multiple Segmentations (1) | 1 | 1 | 1 | 1 | 1 | 1 | 1 | 1 | 1 | 1 |
| ﻿Phantom Study (1) | 0 | 0 | 0 | 0 | 0 | 0 | 0 | 0 | 0 | 0 |
| ﻿Multiple Time points (1) | 0 | 0 | 0 | 0 | 0 | 0 | 0 | 0 | 0 | 0 |
| ﻿Feature Reduction (3) | 3 | 3 | 3 | 3 | 3 | 3 | 3 | 3 | 3 | 3 |
| ﻿NonRadiomics (1) | 1 | 1 | 1 | 0 | 1 | 1 | 1 | 1 | 1 | 1 |
| ﻿Biological Correlates (1) | 1 | 1 | 1 | 0 | 0 | 1 | 1 | 1 | 1 | 1 |
| ﻿Cut-Off (1) | 1 | 1 | 1 | 0 | 0 | 1 | 1 | 1 | 1 | 1 |
| ﻿Discrimination/Resampling (2) | 2 | 2 | 1 | 2 | 2 | 2 | 2 | 1 | 1 | 2 |
| ﻿Calibration/Resampling (2) | 1 | 1 | 1 | 1 | 0 | 1 | 1 | 0 | 1 | 2 |
| ﻿Prospective (7) | 0 | 0 | 0 | 0 | 0 | 0 | 0 | 0 | 0 | 0 |
| ﻿Validation (5) | 4 | 2 | 4 | -5 | 4 | 2 | 3 | 2 | 2 | 2 |
| ﻿Gold Standard (2) | 0 | 2 | 2 | 2 | 0 | 2 | 2 | 2 | 2 | 2 |
| ﻿Clinical Utility (2) | 2 | 2 | 2 | 2 | 2 | 2 | 2 | 0 | 0 | 2 |
| ﻿Cost (1) | 0 | 0 | 0 | 0 | 0 | 0 | 0 | 0 | 0 | 0 |
| ﻿Open Science (4) | 1 | 1 | 2 | 1 | 2 | 2 | 2 | 2 | 2 | 2 |
| Total score (36) | 17 | 18 | 20 | 9 | 16 | 20 | 21 | 14 | 16 | 21 |

Table S3. Individual QUADAS-2 Ratings (LJ F)

| Study | Risk of Bias | | | |  | Applicability Concerns | | |
| --- | --- | --- | --- | --- | --- | --- | --- | --- |
|  | Patient Selection | Index Test | Reference Standard | Flow and Timing |  | Patient Selection | Index Test | Reference Standard |
| Chen et al | Low | Unclear | Low | Low |  | Low | Low | Low |
| Kong et al | Low | Unclear | Low | Low |  | Low | Low | Low |
| Kuang et al | Low | Unclear | High | High |  | Low | Low | Low |
| Mao et al | Low | Unclear | High | High |  | Low | Low | Low |
| Peng et al | Unclear | Low | Low | High |  | Low | Low | Low |
| Zhao et al | Unclear | Unclear | Low | Low |  | Low | Low | Low |
| Liu et al | Unclear | Unclear | Low | Low |  | Low | Low | Low |
| Guo et al | Unclear | Unclear | Low | Low |  | Low | Low | Low |
| Bai et al | Low | Unclear | Low | Low |  | Low | Low | Low |
| Cannella et al | Low | Unclear | Low | Low |  | Low | Low | Low |

Low = Low risk; High = High risk; Unclear = Unclear risk.

Table S4. Individual QUADAS-2 Ratings (LJ H)

| Study | Risk of Bias | | | |  | Applicability Concerns | | |
| --- | --- | --- | --- | --- | --- | --- | --- | --- |
|  | Patient Selection | Index Test | Reference Standard | Flow and Timing |  | Patient Selection | Index Test | Reference Standard |
| Chen et al | Low | Unclear | Low | Low |  | Low | Low | Low |
| Kong et al | Unclear | Unclear | Low | Low |  | Low | Low | Low |
| Kuang et al | Unclear | Unclear | High | High |  | Low | Low | Low |
| Mao et al | Low | High | High | High |  | Low | Low | Low |
| Peng et al | Low | Low | Low | Low |  | Low | Low | Low |
| Zhao et al | Low | Unclear | Low | Low |  | Low | Low | Low |
| Liu et al | Unclear | Unclear | Low | Low |  | Low | Low | Low |
| Guo et al | Unclear | Unclear | Low | Low |  | Low | Low | Low |
| Bai et al | Low | Unclear | Low | Low |  | Low | Low | Low |
| Cannella et al | Low | High | Low | Low |  | Low | Low | Low |

Low = Low risk; High = High risk; Unclear = Unclear risk.

Table S5. Result of QUADAS-2 rating.

| Study | Risk of Bias | | | |  | Applicability Concerns | | |
| --- | --- | --- | --- | --- | --- | --- | --- | --- |
|  | Patient Selection | Index Test | Reference Standard | Flow and Timing |  | Patient Selection | Index Test | Reference Standard |
| Chen et al | Low | Unclear | Low | Low |  | Low | Low | Low |
| Kong et al | Low | Unclear | Low | Low |  | Low | Low | Low |
| Kuang et al | Low | Unclear | High | High |  | Low | Low | Low |
| Mao et al | Low | High | High | High |  | Low | Low | Low |
| Peng et al | Unclear | Low | Low | High |  | Low | Low | Low |
| Zhao et al | Low | Unclear | Low | Low |  | Low | Low | Low |
| Liu et al | Unclear | Unclear | Low | Low |  | Low | Low | Low |
| Guo et al | Unclear | Unclear | Low | Low |  | Low | Low | Low |
| Bai et al | Low | Unclear | Low | Low |  | Low | Low | Low |
| Cannella et al | Low | High | Low | Low |  | Low | Low | Low |

Low = Low risk; High = High risk; Unclear = Unclear risk.
